# Including two-dimensional pressure-focusing in transmission-line cochlear models

**DOI:** 10.1121/10.0044580

**Published:** 2026-08-05

**Authors:** Alessandro Altoè, Christopher A. Shera

**Affiliations:** Department of Otolaryngology, University of Southern California, Los Angeles, California 90033, USA

## Abstract

Transmission-line models are the simplest physics-based cochlear models that include an explicit representation of the traveling wave, which plays an important role for encoding complex sounds in the auditory periphery. Despite their many attractive features, transmission-line models suffer from well-known shortcomings that prevent them from replicating the experimental data or explicating the physical functioning of the cochlea. The main issue is that they neglect important two-dimensional (2D) hydrodynamic effects, widely known as “pressure focusing,” which enhance the vibration of the cochlear sensory tissue in a wavelength-dependent fashion. Here, we present an efficient method for including 2D pressure-focusing in transmission-line models.

## 1. Introduction

Time-domain transmission-line cochlear models have been valuable tools for auditory research. By employing a one-dimensional (1D) approximation of the cochlear physics, this class of models provides a practical means to incorporate an explicit representation of the cochlear traveling wave in models of the auditory periphery. A representation of the traveling wave is important in a variety of scenarios, for example, to study the relation between inner-ear responses and non-invasive auditory-evoked responses (e.g., Neely and Rasetshwane, 2017; Verhulst *et al.*, 2018) or when studying phenomena that involve the spatially distributed nature of cochlear processing (e.g., suppression,Versteegh and van der Heijden, 2013; Charaziak *et al.*, 2020).

One of the most attractive features of transmission-line models is their ability to efficiently perform time-domain simulations. In contrast, more realistic two-dimensional (2D) (e.g., Vencovskỳ *et al.*, 2019) and three-dimensional (3D) models (Meaud and Lemons, 2015) have substantially higher computational complexity. On the other hand, transmission-line models approximate the cochlea in 1D, and by doing so, they neglect important 2D and 3D short-wave hydrodynamic effects that significantly enhance the motion of the sensory tissue (pressure focusing, see Altoè (2026) for a recent review). In order to satisfyingly replicate the basilar membrane (BM) response observed in experiments, 1D transmission-line models compensate for the lack of pressure focusing by enhancing the mechanical tuning of the sensory tissue (Shera *et al.*, 2005). While this approach guarantees that, in theory, the BM response of a *linear* 2D model can be precisely captured by an equivalent transmission line, this equivalence does not hold when the model is made nonlinear or when the model is parametrized to simulate responses in different scenarios (e.g., *in vivo* vs postmortem). In practice, matters are even less ideal because the implementation of a time-domain transmission-line model is further constrained by other requirements, such as stability and causality. As a consequence, current transmission-line models struggle to replicate basic experimental observations, such as the range of active gain (i.e., the difference in BM vibration gain *in vivo* vs postmortem).

Here, we propose a simple but effective method to include 2D short-wave dynamics in time-domain transmission-line models, fixing their shortcomings at a modest computational cost. The key idea is that in a 2D model the gradients of fluid motion over one spatial dimension are entirely determined by the dynamics of fluid in the other (Siebert, 1974): 2D effects currently missed by transmission-line models can be encapsulated in a 1D description of cochlear mechanics. While this idea dates back at least to the 1970s and 1980s (Siebert, 1974; Duifhuis, 1988), its current application requires solving transcendental functions of wavelength, rendering it impractical for time-domain simulation. Here, we demonstrate that the effect of wavelength-dependent 2D fluid dynamics on the motion of the cochlear partition (CP) is mathematically equivalent to a 1D spatial filter, which is easily included in a time-domain transmission-line model. To keep matters simple, we illustrate the method using a linear 2D scaling-symmetric box model, which can be rendered “active” or “passive” by switching the cochlear amplifier on or off.

## 2. Approximating 2D pressure-focusing in a time-domain transmission-line model

**Fig. 1.**
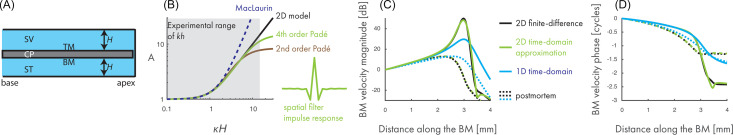
(A) Schematic representation of the 2D box model of the cochlea. The model consists of two identical fluid-filled chambers, SV and ST divided by the CP. The CP is a blob of fluid and tissue delimited on top by the TM and on the bottom by the BM. (B) 2D pressure focusing factor [ A(κ) Eq. (4)] as a function of κH (i.e., the wavenumber κ normalized by scala height *H*). The colored curves represent rational functions that approximate A(κ): the second order Maclaurin series (i.e., Taylor expansion around κ=0), and the second- and fourth-order Padé approximants. The gray area shows the range of interest of κH for a model of the gerbil cochlea—in the gerbil base shortest wavelengths are on the order of 200 *μ*m and the scala height is on the order of 500 *μ*m (Ren, 2002; Plassmann *et al.*, 1987). The green curve in the inset shows the impulse response of the pressure-focusing spatial filter ( α), obtained by inverse Fourier transforming the fourth-order Padé approximant of the function A(κ), which we employed to mimic wavelength-dependent 2D pressure-focusing in a transmission-line model. (C), (D) BM response (magnitude and phase) to a 16 kHz tone, calculated in an active, scaling-symmetric model, using 2 D finite-differences (Neely, 1981), the proposed approximation of 2D pressure-focusing in a time-domain transmission-line model and using the standard 1D approximation (no pressure-focusing, $p_{0}=\bar{p}$). Dashed lines indicate “passive” responses (i.e., responses calculated when turning off the cochlear amplifier in the model).

Consider the simple 2D model of the cochlea depicted in Fig. 1(A). The model is a fluid-filled box of length *L*, partitioned into two scalae—scala vestibuli (SV) and scala tympani (ST)—of equal height (*H)* by the CP, which represents the properties of the BM, organ of Corti, and tectorial membrane (TM). We start by considering the pressure-difference between the scale, where $p(x,z,t)=p_{\text{SV}}(x,z,t)-p_{\text{ST}}(x,-z,t)$. When the cochlea is driven by a pure tone of angular frequency *ω*, we can express the traveling wave as a superposition of stationary waves using the Fourier series

$$p(x,z,\omega ,t)=\sum_{\kappa }P(\kappa )e^{i(\omega t-\kappa x)}\gamma (\kappa ,z),$$ (1)

where P(κ) is the Fourier coefficient of pressure, γ is a function that we will determine below, κ=πj/L with j∈ℕ the spatial frequency, and *i* is the imaginary unit. Here and in the following, we use lowercase symbols to denote functions in the spatial domain and uppercase for their Fourier coefficients. The Laplace equation for the incompressible cochlear fluid requires that

$$\frac{\partial^{2}p}{\partial x^{2}}+\frac{\partial^{2}p}{\partial z^{2}}=0,$$ (2)

and the boundary conditions at the ceiling of the duct impose that ∂p/∂z=0 at z=H. With these constraints it can be verified that (Siebert, 1974)

$$\gamma (\kappa ,z)=\frac{\text{ cosh}[\kappa (H-z)]}{\text{ cosh}(\kappa H)}.$$ (3)

The pressure $p_{0}=p(x,0)$ drives the motion of the CP. It follows from Eq. (3) that $P_{0}(\kappa )=P(\kappa )$ defined in Eq. (1). At a given spatial frequency *κ* and location *x*, the ratio between driving $P_{0}(\kappa )$ and average pressure $\bar{P}(\kappa )=1/H\int_{0}^{H}P(\kappa )\gamma (\kappa ,z)dz$ is (Duifhuis, 1988)

$$A(\kappa )=\frac{P_{0}(\kappa )}{\bar{P}(\kappa )}=\frac{\kappa H}{\text{ tanh}(\kappa H)},$$ (4)

where A(κ) is the pressure-focusing factor.^1^

The inverse Fourier transform of the pressure-focusing factor $\alpha (x)=F^{-1}A(\kappa )$ represents the impulse response of a spatial filter.^2^ Following Fourier theory, we can express the relation between average and driving pressure as

$$p_{0}(x)=\bar{p}⊛\alpha (x),$$ (5)

where ⊛ denotes convolution. Equation (5) shows that the pressure-focusing effect can be accounted for by a 1D linear spatial filter with impulse response α.

Applying Newton's law and mass conservation, we obtain the well-known 1D equation for the average pressure

$$\frac{d^{2}\bar{p}}{dx^{2}}=\sigma a,$$ (6)

with σ=2ρ/H and *a* the acceleration of the CP. The acceleration of the CP at a given location *x* is determined by the driving pressure and by the CP's mechanical properties. In the time domain, this can be generically described by an “oscillator equation”

$$a=f(p_{0})+g(v,y\ldots ),$$ (7)

where *f* and *g* represent functions of pressure and the variables associated with CP motion—e.g., velocity *v* and displacement *y* and any additional state variables that depend on the specific model. The dependence on location *x* is left implicit for notational simplicity. Note that we are not making any assumptions about the linearity or “locality” of the oscillator equation: Eq. (7) can be nonlinear and include state variables associated with motions at different locations.

The system of Eqs. (5)–(7), together with boundary conditions at the basal and apical ends of the cochlea, completely determine the model's solution over time and space. However, the convolution operation in Eq. (5) largely prevents one from calculating the numerical solution efficiently. The standard engineering solution to this problem consists of expressing the convolution operation through an equivalent ordinary differential equation so that the model boils down to a system of equations that can be solved algebraically. This would be a straightforward matter if the function A(κ) was a polynomial or rational function of κ, indeed with reference to Eq. (1),

$$\frac{\partial p}{\partial x}=-i\kappa p.$$ (8)

Because A(κ) is transcendental, we approximate it with a rational function using a Padé approximant of order *M*

$$A_{P,M}(\kappa )\approx \frac{\sum_{m=0}^{M}a_{m}\kappa^{m}}{1+\sum_{m=1}^{M}b_{m}\kappa^{m}}.$$ (9)

Using this approximation and Eq. (8), Eq. (5) can be rewritten as a differential equation

$$p_{0}+\sum_{m=1}^{M}\frac{1}{{\left(-i\right)}^{m}}b_{m}\frac{d^{m}p_{0}}{dx^{m}}=\sum_{m=0}^{M}\frac{1}{{\left(-i\right)}^{m}}a_{m}\frac{d^{m}\bar{p}}{dx^{m}},$$ (10)

where the dependencies on time *t* and location *x* are omitted for notational simplicity. We found that using a fourth-order Padé approximant works reasonably well in the physiological range of κ [see caption of Fig. 1(B)].

We numerically solve the model by discretizing the cochlea into *N* sections of length Δx and using the state space matrix formalism (Elliott *et al.*, 2007). At the *n*th longitudinal location (*x*[*n*]), the equations of motion of the CP involve the driving pressure and the state variables associated with the partition's oscillator equation [Eq. (7)]. For simplicity, let's consider the CP as an array of damped harmonic oscillators (mass *m*[*n*], damping *d*[*n*], and stiffness *k*[*n*]), excited by the driving pressure $p_{0}[n]$. At any location, the state variables are velocity and displacement $x_{n}={\left(v[n],y[n]\right)}^{T}$ (see Elliott *et al.*, 2007)

$$\dot{v}[n]=\frac{1}{m[n]}(p_{0}[n]-d[n]v[n]-k[n]y[n]),$$ (11)

$$\dot{y}[n]=v[n].$$ (12)

We can rewrite these equations for all sections in a compact in matrix form

$$\dot{x}=\text{Ax}+Bp_{0},$$ (13)

where $x={\left[x_{0},x_{1},\ldots ,x_{N}\right]}^{T}$, $p_{0}={\left(p_{0}[0],p_{0}[1],\ldots ,p_{0}[N]\right)}^{T}$, and where A and B are block-diagonal matrices.

Using finite-difference to express spatial derivates in Eq. (6) and standard boundary conditions at the basal and apical ends (see e.g., Elliott *et al.*, 2007; Verhulst *et al.*, 2012) leads to

$$F\bar{p}-a=q,$$ (14)

where **a** is the vector of CP accelerations, $\bar{p}={\left(\bar{p}[0],\ldots ,\bar{p}[N]\right)}^{T}$, **q** is the source vector (when the cochlea is stimulated by airborn sound, $q={\left[q_{0},0,\ldots ,0\right]}^{T}$), and **F** is the tridiagonal finite-difference matrix. The acceleration of the partition can be expressed in matrix form as

$$a=C\dot{x},$$ (15)

where C in this case “selects” the CP velocity variable.

Finally, we express the driving pressure $p_{0}$ as a function of $\bar{p}$ and express such relations in matrix form. The discrete realization, of order 2*j*, of the spatial filter as Eq. (10), can be obtained using finite-difference to approximate spatial derivates

$$e_{n,n-j}p_{0}[n-j]+\cdots +e_{n,n}p_{0}[n]+\cdots +e_{n,n+j}p_{0}[n+j]=d_{n,n-j}\bar{p}[n-j]+\cdots +d_{n,n}\bar{p}[n]+\cdots +d_{n,n+j}\bar{p}[n+j],$$ (16)

where the coefficients $e_{i,j}$ $d_{i,j}$ depend on the spatial filter, the chosen finite-difference approximation, and the discretization step Δx. In our numerical solver, the coefficients are computed by writing the Padé approximant in filter form [Eq. (10)] and using central finite-difference to approximate the spatial derivatives.^3^ Writing Eq. (16) for each section produces a linear system of *N* equations that can be written in matrix form as

$$Ep_{0}=D\bar{p},$$ (17)

where E and D are N×N banded matrices, whose elements are $e_{i,j}$ and $d_{i,j}$, respectively. We can now express the driving pressure as a function of average pressure through

$$p_{0}=E^{-1}D\bar{p},$$ (18)

where $E^{-1}D$ is the matrix representation of the spatial filter.

Finally, Eq. (13) becomes

$$\dot{x}=\text{Ax}+B^{\prime }\bar{p},$$ (19)

with $B^{\prime }=BE^{-1}D$. The model is now completely defined by Eqs. (14), (15), and (19), which together provide the state-space formulation of a transmission-line model (Elliott *et al.*, 2007).

## 3. Testing the method in an active model

We illustrate the implementation and demonstrate the accuracy of the proposed method using a simple scaling-symmetric phenomenological model of the gerbil cochlea. The macro-mechanics of these types of models have been amply documented and described in the literature (see, e.g., Altoè (2026)). The active micro-mechanics of the model are original, presenting a simple mechanistic implementation of the “overturned” model (Altoè *et al.*, 2022). The CP is represented by two plates, the BM at the bottom and the TM at the top; the net transverse velocity of the partition (i.e., the velocity of its center of mass) is $v=(v_{\text{bm}}+v_{\text{tm}})/2$. The mechanics of the BM are represented by a simple harmonic oscillator, in both the active and the passive case

$${\dot{v}}_{\text{bm}}=\frac{p_{0}}{m}-2\zeta \omega_{c}v_{\text{bm}}-\omega_{c}^{2}y_{\text{bm}},$$ (20)

with ζ and $\omega_{c}$ the damping factor and natural frequency of the cochlear oscillators, respectively. The motion of the TM is the sum of the BM motion and an internal motion ( $v_{\text{int}}$) driven by outer hair cells (OHCs) that modulate the distance between TM and BM on a cycle by cycle basis [ $v_{\text{tm}}=v_{\text{bm}}+v_{\text{int}}$]. Because the BM is much stiffer than the TM, we assume the direct effect of OHCs on BM motion is negligible so that $v=v_{\text{bm}}+v_{\text{int}}/2$. We represent this internal motion as a second oscillator driven by BM displacement

$${\dot{v}}_{\text{int}}=-\tau \omega_{c}^{2}y_{\text{bm}}-2\zeta_{\text{int}}\omega_{c}v_{\text{int}}-\omega_{c}^{2}y_{\text{int}},$$ (21)

where τ and $\zeta_{\text{int}}$ are adjustable parameters and where, for simplicity, we assumed that the internal oscillator's natural frequency is the same as that of the BM. The form of this internal oscillator is based on broadly accepted features of cochlear micromechanics: (1) OHC vibration exhibits a low-pass response relative to that of the BM; and (2) at very low frequencies, OHC forces drive the motion of the upper side of the organ of Corti (the TM and reticular lamina) in opposite phase to the BM, whereas this motion becomes progressively more in phase as the frequency approaches the CF, while continuing to lead the BM up to CF (Ren *et al.*, 2016; Dewey *et al.*, 2021). The purpose of this second oscillator is not to create sharply tuned forces—the oscillator is well damped and broadly tuned—but rather to rotate the phase of the internal motion to be consistent with the experimental data and to allow transfer of energy from the partition to the pressure wave in the amplification region (see Altoè *et al.*, 2022). This idea is not new (see, e.g., Nobili and Mammano, 1996). The novelties of our proposal are that the second oscillator represents a simple qualitative description of what has been recently observed with optical coherence tomography and that it is directly coupled to the surrounding fluid via the TM.^4^ Setting τ=0 abolishes OHC active motions and amplification, allowing one to simulate the response of a passive cochlea. Details of the time-domain implementation, which uses four state variables $\left[v_{\text{bm}},y_{\text{bm}},v_{\text{int}},y_{\text{int}}\right]$, can be found in the accompanying matlab scripts.

Figures 1(C) and 1(D) show the model's BM response (magnitude and phase) for active and passive models. The responses are calculated in a finite-difference frequency-domain 2D model (Neely, 1981) and using our 2D approximation in the time-domain. Because the model is scaling-symmetric, only responses at one frequency (16 kHz) are shown—responses to different frequencies are identical, just shifted along the cochlea. These results show that the difference between the proposed time-domain approximation and the finite-difference 2D model is within a couple of decibels in the active case. In the passive case, the error is negligible, and the curves are virtually identical. In the active case, wavelengths are shorter than in the passive case near the best place—this can be noted by the different phase curvatures in Fig. 1(C)—therefore, the error produced by our approximation is likely caused by the increasing inaccuracy of the Padé approximation with the shortening of wavelength [Fig. 1(B)]. Although the proposed approximation is not exact, its error is small for any practical purpose. More importantly, unlike the standard 1D transmission-line approximation [Fig. 1(C)], which completely fails to reproduce the BM response, the proposed method captures it with only a small quantitative error.

All simulations were performed on a 13″ MacBook Air equipped with an Apple M2 chip (Apple Inc., Cupertino, CA). The spatial discretization was Δx=20 μm ( N=576 sections), and the governing equations were integrated in time using an adaptive step-size Runge Kutta method (ode45 in matlab). Using the basic state-space formulation of Elliott *et al.* (2007), we found no significant difference in computational time between the 1D transmission-line model and our 2D approximation. In each case, a 50 ms simulation with a 16 kHz stimulus required approximately 55 s. These simulation times are not particularly fast, as the solver has a computational complexity of $O(N^{2})$ per time step. By adapting the matrix optimization strategy of Pan *et al.* (2015) to our formulation, the computational complexity was reduced to O(N). This resulted in a substantial speed-up: for a 50 ms simulation, the execution time decreased to approximately 7.7 s for the 2D approximation and to about 3.3 s for the 1D model. In our fastest implementation, the 2D approximation takes about two and a half times longer than the 1D model but remains computationally practical.

These execution times should be interpreted in the context of other cochlear models. Our implementation employs adaptive time stepping, in which the step size is controlled by local error estimates and the specified numerical tolerances. Consequently, solutions with higher-frequency content require smaller time steps. Because the present gerbil model uses a 16 kHz stimulus and exhibits higher-frequency transients than comparable human models, its computational cost is correspondingly greater. Taking these factors into account, together with the fact that the implementation is written in matlab rather than optimized compiled code, the performance compares favorably with widely used transmission-line models. For example, on our machine, it takes approximately 3 s for a 50 ms, 4 kHz simulation using the human cochlear model of Verhulst *et al.* (2018) ( N=1000, Δx=35 μm).

## 4. Discussion

We propose a method to mimic 2D pressure focusing within a 1D transmission-line model by incorporating a spatial filter that reproduces the effects of the 2D fluid dynamics. The method accurately mimics these effects, while retaining the computational efficiency of the transmission-line formulation. Although our matlab implementation adds some computational overhead compared with an optimized 1D transmission-line model, it preserves an O(N) computational complexity per time step. The proposed method reduces wavelength-dependent pressure focusing to a 1D spatial filter, whose accuracy depends on the filter order; for the gerbil cochlea, a fourth-order filter provides an accurate approximation. Importantly, because the filter operates directly on the wavelength-dependent fluid coupling, changes in cochlear amplification automatically produce the corresponding changes in pressure focusing. This behavior is demonstrated by the accurate prediction of both active and passive responses in Figs. 1(C) and 1(D), which were obtained using identical models differing only in the amplification parameter ( τ).

Conceptually, the proposed approach is related to Green's function formulations, in which higher-dimensional fluid dynamics are represented through instantaneous longitudinal coupling of the CP motion. However, rather than explicitly evaluating the full Green's function, the coupling is approximated by a low-order spatial filter that can be incorporated into the transmission-line model with O(N) computational complexity, compared with the $O(N^{2})$ cost of conventional Green's function methods (e.g., Allen, 1977; Vencovskỳ *et al.*, 2019). Previous attempts to incorporate 2D and 3D fluid effects into transmission-line models have generally relied on modifying the underlying 1D model. For example, Elliott and Ni (2018) introduced short-range longitudinal coupling (similar in spirit to the approach proposed here), together with modified micromechanics to account for differences in fluid loading between 1D and 3D based on a detailed analysis of their model. Other transmission-line models have compensated for the absence of pressure focusing either by modifying the CP oscillator equation (Shera *et al.*, 2005; Zweig, 2015) or by introducing a second filter that provides an empirical “boost” to CP motion (Deloche *et al.*, 2026). Such model-specific modifications require re-analysis or re-tuning when the model or its operating conditions change. For example, the required mechanical boost depends on wavelength, which in turns depends on cochlear amplification (Ren, 2002; Shera, 2007; Nankali *et al.*, 2022). In contrast, our approach leaves the underlying mechanics unchanged and accounts for 2D fluid dynamics entirely through a model-independent spatial filter, making it readily applicable to arbitrary cochlear models and parameter changes.

One important aspect of the pressure-focusing spatial filter to keep in mind is that it is symmetric (A(κ) and its approximations are even functions) as shown in inset of Fig. 1(B). This means that the pressure-focusing filter affects “forward” traveling waves in the same way it affects “reverse” waves, i.e., the waves traveling towards the base that give rise to the otoacoustic emission (OAE). In a nutshell, inclusion of the pressure-focusing filter in the transmission-line does not introduce asymmetries between forward and reverse waves that would be detrimental to the simulation of OAEs (see Shera and Altoè, 2023).

The method we propose appears to avoid the problem of numerical stability present in existing 1D models. This numerical stability issue has made it hard for transmission-line models to fully capture the range of BM amplification seen in animal experiments (see Neely and Rasetshwane, 2017; Deloche *et al.*, 2026). In our simulations [Fig. 1(C)] we have “over-amplified” the cochlea a bit to show that the time-domain model is stable with about 50 dB of “active gain” at the characteristic place (typically around 40 dB in gerbil, see Dong and Olson, 2013). We can crank up the gain much more and obtain numerically stable simulations, even up to 90 dB of active gain. We cannot pinpoint the exact reason why our model is so much more stable than models based on classic 1D transmission lines: there are too many differences between our and previous models, including the CP oscillator equation, species-dependent parameters that regulate the propagation of the traveling wave, and possibly different strategies to avoid internal wave scattering that can lead to instability (Elliott *et al.*, 2007). Nonetheless, we note that the high BM gain in our model arises from the contribution of a moderately tuned (highly stable) active oscillator and a very stable spatial filter [Fig. 1(B)]. In contrast, compensating for the lack of pressure focusing by artificially enhancing the CP mechanics tends to make the CP oscillator more sharply tuned than necessary (see, Fig. 15 in Shera *et al.*, 2005), likely pushing the model towards instability.

## 5. Conclusion

We have proposed a method that substantially extends the capabilities of time-domain transmission-line models, enabling them to accurately capture the hydrodynamic mechanism of pressure focusing present in more realistic 2D models while preserving the computational practicality of the transmission-line formulation. Because transmission-line models find applications ranging from computational auditory models to AI-driven hearing-aid algorithms (Drakopoulos and Verhulst, 2023), overcoming their long-standing shortcomings in predicting cochlear mechanical responses while retaining their computational advantages has broad practical relevance.

We illustrated the proposed method using a simple, scaling-symmetric linear box model, and the model code is freely available at https://github.com/AuditoryPhysicsGroup. The proposed methodology can be applied to tapered nonlinear models, including those with the micromechanical irregularities responsible for reflection-type OAEs. A computationally efficient implementation of these more advanced models is left for future work.

## Data Availability

The data that support the findings of this study are openly available in GitHub at https://github.com/AuditoryPhysicsGroup.

## Footnotes

^1^
Note that our treatment of pressure focusing differs from that presented in previous studies, which typically employ the WKB approximation (see, e.g., Shera *et al.*, 2005; Frost, 2024; Altoè, 2026). While in previous treatments the traveling wave is described through a complex wavenumber that is a function of longitudinal location *x*, the same wave is here decomposed into the superposition of sinusoidal functions. The difference between previous and present work is analogous to that between Hilbert and Fourier analysis: whereas a signal is described in terms of time-dependent (complex) instantaneous frequency in the former, in the latter the same signal is described as the superposition of sinusoidal functions of fixed frequency.

^2^
The reader may note that A(κ)→∞ when |κ|→∞. Consequently, its inverse Fourier transform does not exist as a finite ordinary function. For the moment, we proceed formally; the validity of this approach will be clarified shortly.

^3^
The resulting formula is cumbersome; the coefficients are numerically calculated in the script provided at https://github.com/AuditoryPhysicsGroup.

^4^
In classic models, $v=v_{\text{bm}}$, and the second oscillator acts entirely by forcing the BM, with the TM motion not directly coupled to the scala fluid (e.g., Neely and Kim, 1986; Nobili and Mammano, 1996). In contrast, in our model, the TM motion is directly coupled to the scala fluid through $v=(v_{\text{bm}}+v_{\text{tm}})/2$, and the second oscillator acts entirely on the TM.
